# A Novel SPR Immunosensor Based on Dual Signal Amplification Strategy for Detection of SARS-CoV-2 Nucleocapsid Protein

**DOI:** 10.3390/bios13050549

**Published:** 2023-05-15

**Authors:** Lirui Fan, Bin Du, Fubin Pei, Wei Hu, Shasha Feng, Bing Liu, Zhaoyang Tong, Wenyuan Tan, Xihui Mu

**Affiliations:** 1 State Key Laboratory of NBC Protection for Civilian, Beijing 102205, China; fanlirui0813@163.com (L.F.); dubin51979@163.com (B.D.); peifubin@163.com (F.P.); hw270816@163.com (W.H.); fengshasha0319@163.com (S.F.); lbfhyjy@sohu.com (B.L.); billzytong@126.com (Z.T.); 2 School of Chemical Engineering, Sichuan University of Science and Engineering, Zigong 643000, China

**Keywords:** surface plasmon resonance, graphene oxide, Au@Ag@Au nanoparticles, SARS-CoV-2 N protein

## Abstract

Since the global outbreak of coronavirus disease 2019 (COVID-19), it has spread rapidly around the world. The nucleocapsid (N) protein is one of the most abundant SARS-CoV-2 proteins. Therefore, a sensitive and effective detection method for SARS-CoV-2 N protein is the focus of research. Here, we developed a surface plasmon resonance (SPR) biosensor based on the dual signal-amplification strategy of Au@Ag@Au nanoparticles (NPs) and graphene oxide (GO). Additionally, a sandwich immunoassay was utilized to sensitively and efficiently detect SARS-CoV-2 N protein. On the one hand, Au@Ag@Au NPs have a high refractive index and the capability to electromagnetically couple with the plasma waves propagating on the surface of gold film, which are harnessed for amplifying the SPR response signal. On the other hand, GO, which has the large specific surface area and the abundant oxygen-containing functional groups, could provide unique light absorption bands that can enhance plasmonic coupling to further amplify the SPR response signal. The proposed biosensor could efficiently detect SARS-CoV-2 N protein for 15 min and the detection limit for SARS-CoV-2 N protein was 0.083 ng/mL, with a linear range of 0.1 ng/mL~1000 ng/mL. This novel method can meet the analytical requirements of artificial saliva simulated samples, and the developed biosensor had a good anti-interference capability.

## 1. Introduction

Coronavirus disease 2019 (COVID-19) has spread to the whole world, becoming one of the world’s grievous public health problems [1]. SARS-CoV-2, severe acute respiratory syndrome coronavirus 2, mainly spreads in people and causes cough, fever, headache, body pain and other symptoms [2]. Additionally, the nucleocapsid (N) protein is one of the most abundant SARS-CoV-2 proteins, with vigorous immunogenicity and is highly expressed during infection [3]. Furthermore, SARS-CoV-2 N protein is an extremely important candidate protein for the development of various diagnostic methods for novel coronavirus diseases [4]. At present, there are some reliable analytical methods for SARS-CoV-2 N protein such as chemiluminescence immunoassays [5], enzyme-linked immunosorbent assays (ELISAs) [6], lateral flow immunoassays (LFAs) [7] and biosensing immunoassays [8]. Biosensor can realize qualitative analysis and quantitative detection with its advantages of high sensitivity and strong specificity. In particular, surface plasmon resonance (SPR) biosensors [9,10,11] have the multiple merits [12] including real-time monitoring interactions between antigen--antibodies, being label free, and having high sensitivity, strong specificity and reliability. SPR is a coherent oscillation of surface-conducted electrons driven by photons, which could exist on the surface of the metal film [13]. Due to the enhancement of the light–matter interaction, the small refractive index change of the dielectric near the interface could change the SPR coupling condition, which could obtain a change in one of the parameters of the measured light, such as wavelength [14]. The principle of SPR sensors is based on the measurement of the resonance wavelength changes reflecting the interaction between the target analyte and the ligand molecules [15]. Additionally, SPR sensors have been reported for broad applications in clinical diagnosis [15], biochemistry [16], food safety analysis [17] and environmental monitoring [18]. A number of papers have been published on the detection of SARS-CoV-2 based on SPR biosensors [19,20,21]. For example, Chen et al. [22] exploited thiol-modified niobium carbide MXene quantum dots (Nb2C-SH QDs) as a signal capture probe which could capture N-gene-targeted aptamers to detect the SARS-CoV-2 N gene with a detection limit of 4.9 pg/mL. Wu et al. [23] reported a sandwich structure method for the detection of SARS-CoV-2 spike S1 protein with a detection limit of 12 fg/mL. Uddin et al. [24] had analyzed an SPR biosensor for the detection of SARS-CoV-2 and conducted extensive data simulation for structural optimization and performance analysis. Therefore, it is possible to utilize SPR biosensors to sensitively and efficiently detect SARS-CoV-2 based on these studies.

Nanomaterials [25,26,27] have attracted a lot of interest in the field of SPR biosensors based on their advantages of unique optical and electronic properties, especially noble metal nanoparticles and 2D nanomaterials. Noble metal nanoparticles are employed as ideal SPR biosensor signal amplification materials, especially gold nanoparticles (Au NPs) [28] and silver nanoparticles (Ag NPs) [29]. The reasons for amplification ascribed to their higher surface-to-volume ratio, higher refractive index and electromagnetically coupling interaction between noble metal nanoparticles and plasma waves propagating on the surface of the gold film. Several literatures have been reported on the enhancement of SPR biosensors based on noble metal nanoparticles. For example, Yi et al. [15] developed a gold–silver alloy film for the detection of cancer antigen 125 by sandwich immunoassay with a detection limit of 0.8 ng/mL, which was two orders of magnitude lower than that of traditional detection methods. Gu et al. [30] reported a novel SPR sensor for microRNA 141 detection based on the Au@Ag NRs amplification strategy. Additionally, the proposed biosensor exhibited high sensitivity with a detection limit of 50 aM. However, there are few studies on the enhancement of SPR sensors based on Au@Ag@Au NPs. Additionally, Au@Ag@Au NPs were mostly utilized to enhance Raman scattering response signals [31,32], indicating that Au@Ag@Au NPs may enhance plasmon coupling [33,34]. Therefore, it is possible to enhance SPR response signals based on Au@Ag@Au NPs. Additionally, the effectively immobilized ligand molecules on the surface of the sensing chip is crucial in development for high sensitivity of SPR biosensors. Consequently, nanomaterials could be introduced to improve the amount of immobilized ligand molecules on the surface of sensor chip. Recently, 2D nanomaterials, such as graphene oxide (GO) [35,36,37] with its advantages of a large specific surface area, an abundance of oxygen-containing functional groups, and excellent biocompatibility, have attracted enormous attention in enhancing the sensitivity of SPR sensors. GO could provide the unique optical absorption band that enhances plasma coupling and could be chemically modified to immobilize more ligand molecules. Therefore, GO is an ideal material for the construction of novel SPR biosensors. Nevertheless, there are few reports on SPR biosensing detection based on the dual signal-amplification effect of noble metal complexes and 2D nanomaterials.

In this work, we developed a novel SPR biosensor based on the Au@Ag@Au NPs and GO dual signal amplification strategy for the quantitative detection of SARS-CoV-2 N protein. Au@Ag@Au NPs exhibits advantages in the enhanced SPR response signal such as high refractive index and the capability to electromagnetically couple with plasma waves propagating on the surface of the gold film. Meanwhile, GO displays benefits such as a large specific surface area, abundant oxygen-containing functional groups on the surface and unique light absorption bands that can enhance plasmonic coupling. Therefore, the capture antibody Ab_1_ was immobilized on the surface of a GO-modified gold film by EDC coupling. Additionally, nanoconjugates (Au@Ag@Au/Ab_2_), obtained by coupling detection antibody Ab_2_ to Au@Ag@Au NPs, were employed as a signal amplification probe, and enriched SARS-CoV-2 N protein were combined in a sample chamber to form an Ab1/SARS-CoV-2 N protein/Ab_2_-Au@Ag@Au sandwich structure. The developed SPR biosensor expands the new road for highly sensitive detection of the novel coronavirus.

## 2. Experimental Procedure

Details of the reagents, instruments and construction of the SPR sensor can be found in the Supplementary Materials.

**Figure 1:**
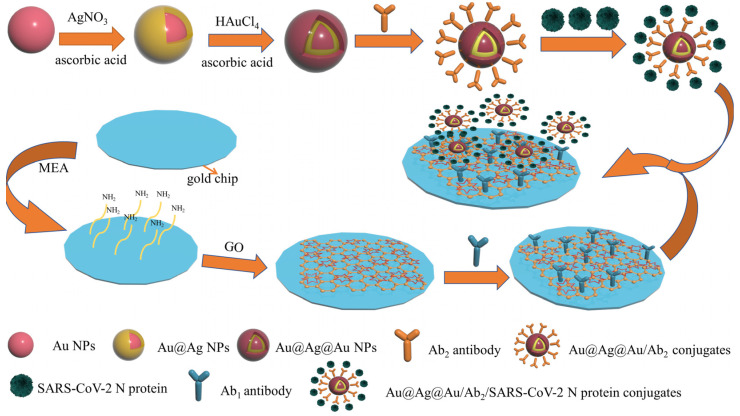
The schematic diagram for detecting SARS-CoV-2 N protein based on SPR sensor with Au@Ag@Au NPs and GO amplification strategy.

In this study, an SPR biosensing platform based on the Au-MEA-GOs/Ab_1_ sensing film and combined with Au@Ag@Au/Ab_2_ sandwich method was constructed for the detection of SARS-CoV-2 N protein (Figure 1). First at all, gold nanoparticles were obtained by reducing chloroauric acid with sodium citrate. Secondly, the silver shell layer was formed on the surface of the gold nanospheres making use of the reduction of silver nitrate by ascorbic acid; in order to protect the silver nanoparticles from oxidation, Au@Ag@Au NPs with a core–shell structure were formed on the surface by the reduction of chloroauric acid using ascorbic acid. It has been shown that Au@Ag@Au NPs are capable of forming Au@Ag@Au/Ab_2_ complexes by coupling with antibody Ab_2_ at pH 8.0 at room temperature [38]. The Au@Ag@Au/Ab_2_ complex was bound to the SARS-CoV-2 N protein for further use. The clean new gold chip was modified with amino groups by immersion to make the gold chip positively charged; the gold chip was modified with graphene oxide through electrostatic interactions, and antibody Ab_1_ was immobilized on the surface of the gold chip through the amide bonds formed by the abundant carboxyl groups on the surface of graphene oxide and the amino groups on antibody Ab_1_. The gold chip modified with antibody Ab_1_ was then fixed onto the SPR prism through the sample chamber and injected with Au@Ag@Au/Ab_2_ complexes conjugated to the SARS-CoV-2 N protein for sensing detection.

### 2.1. Synthesis of Au@Ag@Au NPs

Au NPs were prepared by Yang’s method [39]. Briefly, 3 mL of 1 wt % HAuCl_4_ solution was added into 300 mL of Milli-Q water (18.2 MΩ cm) in a 500 mL round-bottom flask with vigorous stirring. When the solution was heated to 100 °C, 2.25 mL of a 1% aqueous solution of sodium citrate was injected into the boiling solution. The solution was heated in an oil bath at 100 °C for 30 min and condensed through condenser tube at the same time. After natural cooling to room temperature, the colloids were stored at 4 °C for further use.

The Au@Ag@Au core–shell nanospheres was prepared according to the Fales’s method [40]. In brief, 2 mL of a 0.05 M aqueous solution of AgNO_3_ and equivalent 0.1 M ascorbic acid solution were introduced into 100 mL of prepared colloids with vigorous stirring. After 30 min, 2 mL of 0.1 M ascorbic acid was added and then incubated for 30 min, and finally 1 mL of HAuCl_4_ solution was injected in the as-prepared Au@Ag@Au precursor solution for 30 min.

### 2.2. Preparation of Sensing Films Based on GO

The new gold chips were ultrasonically cleaned using acetone and anhydrous ethanol for 30 min each. The clean gold chips were dried in a nitrogen gas stream. The MEA aqueous solution at a concentration of 7 mg/mL was used to modify the clean gold chip at 25 °C for 30 min. Then, the chip was immersed in GO solution for 5 h to fix the GO onto the surface of the gold chip at 25 °C. Additionally, the carboxyl functional group on the GO-modified chip was activated by EDC/NHS (10 mg/mL, 1:1 (*v*/*v*)) for one hour. Additionally, 200 μL Ab_1_ was dropped onto the surface of the chip at 25 °C for 12 h. Finally, an ethanolamine solution (1 mol/L, pH = 8.5) was injected to block unbound carboxyl functional group at 25 °C for one hour.

### 2.3. Functionalization of Au@Ag@Au NPs

Initially, 1 mL Au@Ag@Au NPs solution was adjusted a pH of 8.2–8.4 using a 1 M NaOH solution. The certain concentration of antibody Ab_2_ against SARS-CoV-2 N protein was mixed with the Au@Ag@Au solution with the certain concentration with shaking for 1 h at room temperature. Then, 10% BSA was added and the mixture was incubated for 1 h to block the unbound sites on the Au@Ag@Au NPs. Afterward, the Au@Ag@Au/Ab_2_ solution was purified by centrifugation at 7000× *g* for 3 min to remove any Ab_2_ not bound to nanospheres. Subsequently, the Au@Ag@Au/Ab_2_ conjugates were redispersed in 1 mL PBS buffer containing 1% BSA.

### 2.4. Immunoassay of Sensing Films Based on GO for SARS-CoV-2 N Protein

Primarily, 800μL of SARS-CoV-2 N protein with various concentrations (0.1, 1, 10, 50, 500, and 1000 ng/mL) were added into 200 μL nanoconjugates Au@Ag@Au/Ab2. Afterward, the Au@Ag@Au/Ab_2_/SARS-CoV-2 N protein nanoconjugates were incubated at room temperature for 1 h, washed by centrifugation at 3000× *g* for 5 min to remove non-specifically bound SARS-CoV-2 N protein and redispersed in 1 mL PBS buffer containing 1% BSA. Finally, 200 μL Au@Ag@Au/Ab_2_/SARS-CoV-2 N protein conjugates were injected into sample chamber on the Ab_1_/GO-modified gold chip to carry out sensing detection for 15 min and the 1% BSA PBS buffer was used as blank sample.

### 2.5. Specificity and Stability

The sensing chip with immobilized antibody Ab_1_ was prepared and stored in PBS buffer solution at 4 °C. The sensing chip was tested every 7 days and fixed on an SPR prism with a drop of diiodomethane, injected with 200 μL of PBS buffer solution, and the SPR response signal was recorded to obtain the corresponding resonance wavelength λ_k_ after the resonance curve was stabilized. After injecting 200 μL of SARS-CoV-2 N protein containing the same concentration (500 ng/mL) and detecting for 15 min, the SPR response signal was recorded and the corresponding resonance wavelength λ_l_ was obtained for 42 consecutive days to investigate the stability of the Au-MEA-GOs/Ab_1_-based sensing chip. N protein of influenza A (Flu A), N protein of hemagglutinin of influenza B (Flu B), respiratory syncytial virus attachment protein G (RSV), N protein of middle east respiratory syndrome coronavirus (MERS), bovine serum albumin (BSA), lysozyme and immunoglobulins, were enriched and separated by the Au@Ag@Au/Ab_2_ nanoconjugates, and put into the sample chamber for SPR sensing to measure the changes in the resonance curve and to test the specificity of the method.

### 2.6. Preparation of Spiked Saliva Samples

The saliva samples were diluted by PBS buffer solution (1:50), and the standards of SARS-CoV-2 N protein were added to the diluted solution to obtain the final concentrations of 0.2 ng/mL, 100 ng/mL and 400 ng/mL, and the same concentration was repeated three times to compare the recoveries.

## 3. Results and Discussion

### 3.1. Characterization of Au@Ag@Au NPs

**Figure 2:**
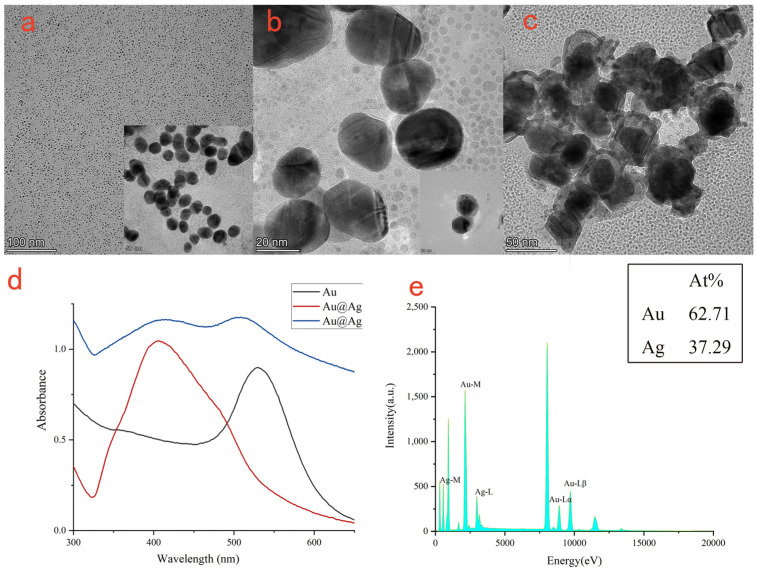
TEM picture of Au NPs (**a**); TEM picture of Au@Ag NPs (**b**); TEM picture of Au@Ag@Au NPs (**c**); the UV-vis absorption spectra of Au NPs, Au@Ag NPs, Au@Ag@Au NPs (**d**); the EDS spectra of Au@Ag@Au NPs (**e**).

The morphologies and sizes of Au NPs (Figure 2a), Au@Ag NPs (Figure 2b), Au@Ag@Au NPs (Figure 2c) were confirmed by TEM. Firstly, sodium citrate was used to reduce chloroauric acid to form gold nanoparticles with a diameter of about 25 nm. Then, the same amount of ascorbic acid and silver nitrate were added to the synthesized gold nanoparticle solution to reduce the silver nitrate solution on the surface of AuNPs using the ascorbic acid to form Au@Ag NPs with a core–shell structure and a particle size of about 29 nm. Then, ascorbic acid and chloroauric acid were added to the solution of the synthesized Au@ Ag NPs; on the surface of Au@Ag NPs, ascorbic acid was reduced to chloroauric acid to form Au@Ag@Au NPs with a core–shell structure and a particle size of about 47 nm.

Core-shell nanoparticles such as Au@Ag NPs usually have adjustable SPR characteristics, good enhancement and stability. Their optical properties are affected by the composition of the core–shell materials, and the SPR effects can be regulated by changing the composition or structure of the shell or core. As shown in Figure 2d, pure gold nanoparticles had a UV-vis absorption peak at ~529 nm, and the UV-vis absorption peak blue shifted to ~406 nm after coating it with silver shell. When Au@Ag NPs are further coated with gold nanoparticles, Au@Ag@Au NPs were formed, and the UV-vis absorption peaks appeared as double shoulder peaks at ~520 nm and ~406 nm. The UV-vis absorption peak generated by the gold nanoparticles (~520 nm) was slightly higher than that generated by the silver nanoparticles (~406 nm), indicating that the outermost Au shell had been coated. Core–shell nanoparticles such as Au@Ag@Au NPs had similar optical properties to bare gold or silver nanoparticles, but the enhanced intensity was higher.

The EDS measurement was used to analyze the composition of the Au@Ag@Au NPs (Figure 2e). It could be observed that the contents of Au and Ag elements were 62.71 wt % and 37.29 wt %, respectively, indicating that the synthetic materials contained Au and Ag elements.

### 3.2. Functionalization of Au@Ag@Au NPs

**Figure 3:**
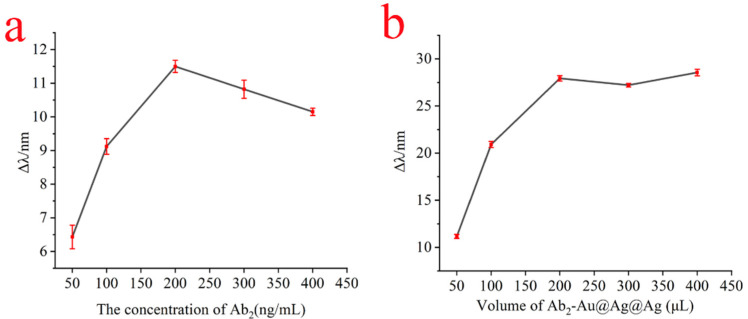
The relationship between the shift in the resonance wavelength and the concentration of antibody Ab_2_ (**a**); the relationship between the shift in the resonance wavelength and the volume of the Au@Ag@Au/Ab_2_ conjugates (**b**).

The Au@Ag@Au NPs were coupled to the antibody Ab_2_ to form a signal amplification probe at pH 8.0. In particular, the concentration of antibody Ab_2_ modification on Au@Ag@Au NPs composites was critical to whether the Au@Ag@Au NPs composites were effective in enhancing the sensitivity of SPR sensors. Too low of a concentration of antibody Ab_2_ would lead to inability to detect high concentrations of the sample to be tested, and too high of a concentration of antibody Ab_2_ would lead to antibody waste. Therefore, in order to estimate the immobilization amount of antibody Ab_2_ to couple on the surface of Au@Ag@Au NPs, the immobilized amount of Ab_2_ was quantitatively estimated according to the absorbance of the Ab_2_ solution after incubation. As shown in Table S1, it was obviously observed that the absorbance increased gradually, indicating antibody Ab2 coupled at Au@Ag@Au NPs surface up to saturated when the concentration of antibody Ab2 was greater than 200 ng/mL. Therefore, 200 ng/mL was chosen as the ideal concentration. At the same time, the relationship between the variation in resonance wavelength and the concentration of antibody Ab_2_ was established. As shown in Figure 3a, when the antibody Ab_2_ concentration was in the range of 50 ng/mL to 200 ng/mL, the amount of change in resonance wavelength increased with the increase in antibody Ab_2_ concentration; when the antibody Ab_2_ concentration was 200 ng/mL, the binding sites on the surface of Au@Ag@Au NPs were almost completely occupied by antibody Ab_2_, and the maximum response signal for SPR detection was obtained, which was reflected by the maximum resonance shift of 11.5 nm. When the concentration of antibody Ab_2_ continued to increase, it led to an increase in spatial site resistance, which in turn led to a decrease in the amount of SPR resonance shift change. Taking into account the consumption of the antibody, the optimal amount of antibody Ab_2_ to be used was 200 ng/mL.

In immunoassays, the volume of Au@Ag@Au/Ab_2_ coupling was critical for the intensity of the enhanced SPR signal of the Au@Ag@Au NPs composites. As shown in Figure 3b, it can be clearly observed that the amount of resonance curve variation increases with the volume of the coupling compound in the range of 0.5 mL to 2 mL for the Au@Ag@Au NPs composites; when the volume of the coupling compound was greater than 2 mL, the amount of resonance curve variation remained basically constant and reached the maximum displacement variation, in which the maximum resonance shifted about 27 nm.

### 3.3. Fabrication and Characterization of Biosensing Film

The prepared good sensor chip could not only affect the sensitivity of sensor but also the cost. Therefore, the amount of MEA, the amount of graphene oxide, and the amount of antibody Ab_1_ were considered.

**Figure 4:**
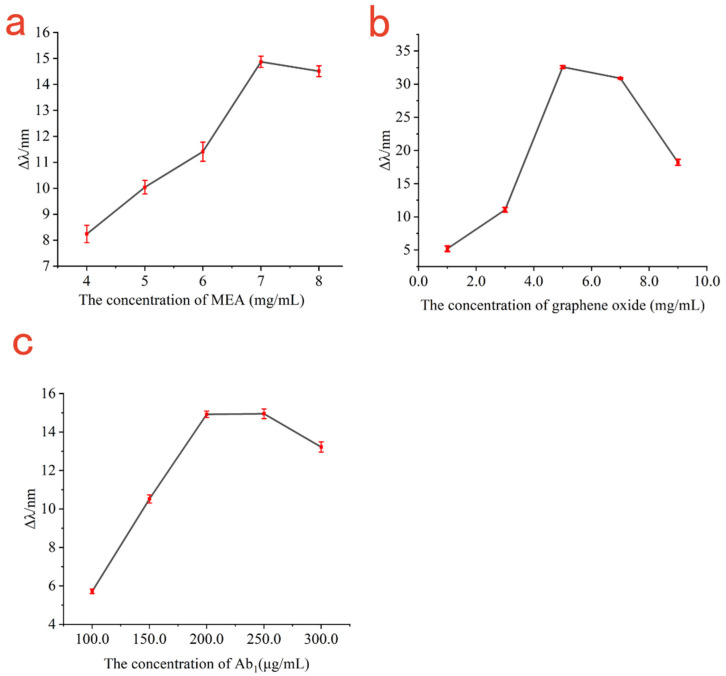
The relationship curve between the shift in resonance wavelength and the concentration of MEA (**a**); the relationship between the shift in resonance wavelength and the concentration of graphene oxide (**b**); the relationship between the shift in resonance wavelength and the concentration of antibody Ab_1_ (**c**).

The amount of MEA directly affected the amount of immobilized GO. Therefore, the relationship curve between the amount of resonance wavelength changes and the concentration of MEA was established. As shown in Figure 4a, with the enhancement of MEA concentration, the amount of changes in resonance wavelength gradually increased until the concentration of MEA ≥ 7 mg/mL, at which the amount of change of resonance wavelength was almost maintained at about 14 nm; increasing the concentration of MEA further did not change resonance wavelength. If the concentration of MEA was too high or the reaction time of MEA on the gold chips was too long, it would make the gold chips become transparent and would affect the SPR resonance curve. Therefore, the optimal concentration of MEA was 7 mg/mL considering the consumption of MEA.

The concentration of GO not only determines the amount of immobilized antibody Ab_1_, but also adjusts the transmittance. Therefore, the relationship curve between the amount of resonance wavelength changes and graphene oxide concentration was established. As shown in Figure 4b, the amount of changes in resonance wavelength increased with the concentration of GO in the range of 1 mg/mL to 5 mg/mL. When the concentration of GO was 5 mg/mL, the surface plasmon resonance effect was clearest, resulting in the incident light being almost completely absorbed by the enhanced surface plasmon resonance to obtain the maximum shift of 32.59 nm. When the concentration of GO was greater than 5 mg/mL, the resonance wavelength gradually decreased with the increase in graphene oxide concentration. Therefore, the optimal concentration of graphene oxide was 5 mg/mL.

In immunoassays using SPR sensors, the concentration of antibody immobilized on the sensing gold film is critical for effective detection of the antigen. If the antibody concentration is too low, the surface sensing chip may not to be fully covered by antibodies, resulting in more exposed binding sites and the possibility of increasing non-specific adsorption; it can also lead to the failure to detect higher concentration of the sample to be measured. However, if the antibody concentration is too high, it would lead to antibody waste. Therefore, the relationship curve between the variation of the resonance wavelength and the concentration of antibody Ab_1_ was established. As shown in Figure 4c, when the antibody Ab_1_ concentration was in the range of 100 μg/mL to 200 μg/mL, the amount of resonance wavelength change increased with the increase in antibody Ab_1_ concentration; when the antibody Ab_1_ concentration was 200 μg/mL, the surface of the sensing chip was almost completely occupied by antibody Ab_1_ to obtain the maximum response signal for SPR detection, which the resonance shift of about 14.92 nm. Taking into account the consumption of the antibody, the optimal concentration of antibody Ab_1_ was 200 μg/mL.

**Figure 5:**
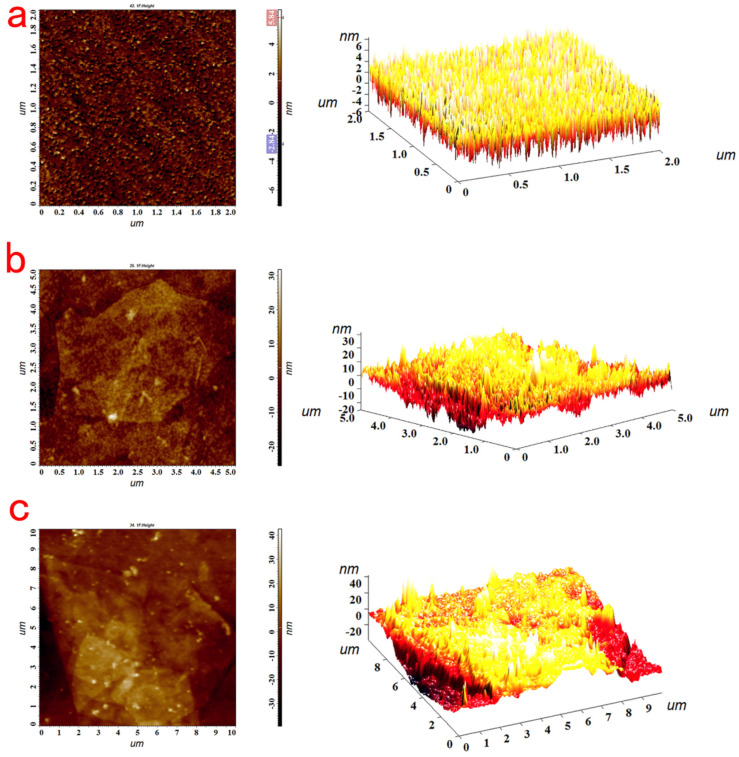
The AFM picture of the bare film (**a**); the AFM picture of the GO immobilized on the surface of the chip (**b**); the AFM picture of Ab1−GO binding to the surface of the chip (**c**).

Figure 5a shows a large number of uniformly distributed small particles in the AFM image of the bare film, and its root mean square (RMS) surface roughness was 1.286 nm, suggesting that the bare gold film was smooth. After the clean bare gold chip was immersed in 7 mg/mL MEA aqueous solution for 30 min, the sensing chip obtained the positive charge through the gold thiol bonds that conjugate between the thiol group in MEA and the gold on the surface of the sensing chip. Then, the positively charged gold chip was immersed in a 5 mg/mL graphene oxide aqueous solution for 5 h, so that the gold chip was modified with graphene oxide. Compared with Figure 5a, it can be clearly observed in Figure 5b that graphene oxide expanded on the side through Π-Π conjugation and hydrophobic interactions to stack into sheets. The root mean square (RMS) surface roughness was 5.983 nm, and the surface roughness of the gold sheet was increased, which proves that graphene oxide was successfully modified onto the surface of the gold chip. Since a monolayer of graphene oxide can absorb light, it also has a high transmittance; when the graphene oxide was modified to the surface of the gold chip, the high transmittance of the graphene oxide could ensure that the evanescent wave could propagate to the surface of the object for detection when the SPR effect occurs. Therefore, graphene oxide can absorb more incident light energy to maintain resonance. In addition, graphene oxide has a large specific surface area, which can enhance the change in refractive index of the sensing surface. At the same time, the surface of graphene oxide is rich in carboxyl groups, and the carboxyl group is activated by EDC/NHS. The carboxyl group on graphene and the amino group of Ab_1_ antibody were used to immobilize the Ab_1_ antibody on the surface of the gold chip through amide bonds. As shown in Figure 5c, compared with the AFM image only modified with graphene oxide, the surface of the gold chip modified with the Ab_1_ antibody was rougher, with a roughness of 10.234 nm, proving that the Ab_1_ antibody was successfully immobilized on the surface of the gold chip.

### 3.4. Au@Ag@Au NPs Enhanced SPR Sensing for Detection of SARS-CoV-2 N Protein

**Figure 6:**
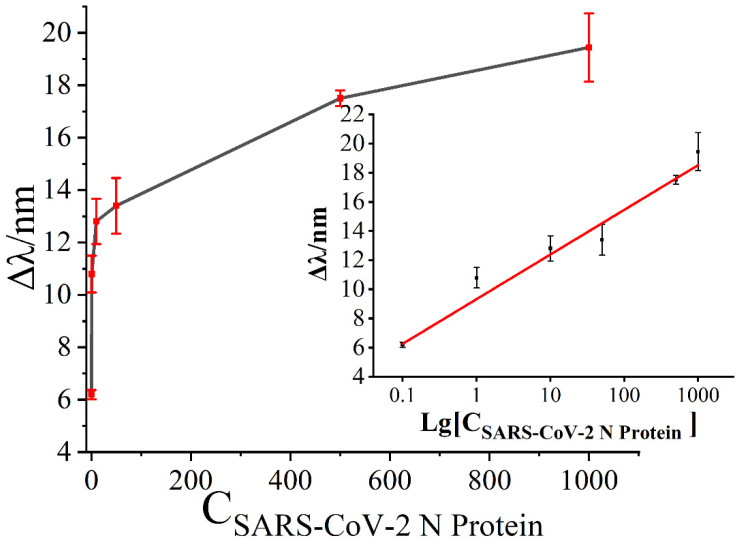
The relationship between the shift in resonance wavelength and the concentration of SARS-CoV-2 N protein.

To evaluate the sensitivity of the SPR biosensor, various concentrations of SARS-CoV-2 N protein were tested under the optimal detection conditions. As shown in Figure 6, the changes in resonance wavelength gradually increased with the increase in concentration of SARS-CoV-2 N protein. When the concentration of SARS-CoV-2 N protein was 0.1 ng/mL, the minimum resonance wavelength shifts was about 6.19 nm. Similarly, when the concentration of SARS-CoV-2 N protein was 1000 ng/mL, the maximum resonance wavelength shifts was about 19.44 nm. A linear relationship was observed between the amount of change in resonance wavelength and Lg [C_SARS-CoV-2 N protein_]. Additionally, there was good linearity in the range of 0.1 ng/mL~1000 ng/mL. Based on the theory of S/N ≥ 3, the limit of detection (LOD) was calculated as approximately 0.083 ng/mL (LOD = 3 × standard deviation/slope).

As shown in Table 1, this method has the lowest detection limit compared to other methods for the detection of SARS-CoV-2 N protein.

### 3.5. Stability and Specificity

**Figure 7:**
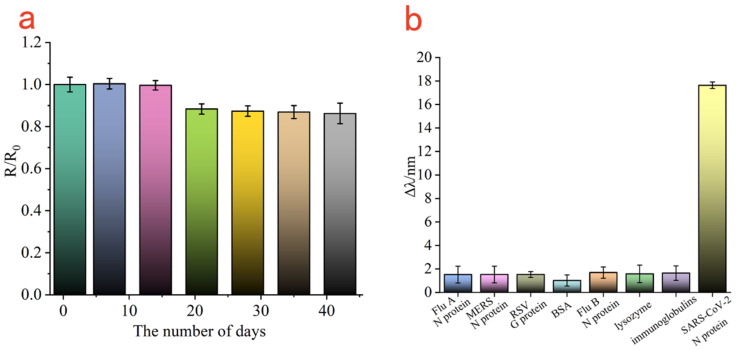
The stored stability of GOs/Ab_1_-modified Au film (**a**); specificity analysis of SPR biosensors (**b**).

To evaluate the storage stability of the Au-MEA-GO/Ab_1_ modified sensing film, the prepared gold chips modified with Au/MEA/GO/Ab_1_ were immersed in PBS buffer and stored at 4 °C. 500 ng/mL SARS-CoV-2 N protein was tested every 7 days and the amount of change in the resonance curve was recorded. The change in the SPR resonance curve detected on the first day was recorded as R_0_, and the change in the SPR resonance curve on the next 6 days was recorded as R. Therefore, the relationship between R/R_0_ and the number of days was established. As shown in Figure 7a, all SPR response values were above 85% within 42 days, indicating the good stability of this sensing gold chip.

To further monitor the specificity of the SPR sensor, the Au-MEA-GOs/Ab_1_ sensing chip was fixed on the SPR prism, and 500 ng/mL of Flu A, Flu B, RSV, MERS, BSA, lysozyme and immunoglobulins were coupled with Au@Ag@Au/Ab_2_ and injected into the sample chamber, and the amount of change in the SPR resonance curves of the different samples was obtained. As shown in Figure 7b, the change in resonance wavelength of SARS-CoV-2 N protein was about 17.6 nm, while the change in resonance wavelength of other analogues were maintained around 1.5 nm. Therefore, it was shown that the biosensor based on the Au-MEA-GOs/Ab_1_ sensing chip and combined with Au@Ag@Au/Ab_2_ sandwich method has good specificity for SARS-CoV-2 N protein.

### 3.6. Analysis of Spiked Saliva Sample

The reliability, feasibility and application of the established biosensor in real samples was evaluated by artificial saliva sample. Artificial saliva sample spiked by SARS-CoV-2 N protein with different spiked levels (0.2 ng/mL, 100 ng/mL, 400 ng/mL) were examined by sandwich method. The experimental results were listed in Table 2. The obtain recoveries ranged from 99.76%~111.13%, and the RSD < 3.88%, which indicates that the developed SPR sensor based on Au@Ag@Au NPs and GO had good reliability, feasibility and application for real sample analysis.

## 4. Conclusions

In summary, taking advantage of the attractive optical properties of Au@Ag@Au NPs including their high refractive index and the ability to electromagnetically couple with plasma waves, and the merits of GO such as a large specific surface area and providing a unique light absorption band, a novel SPR biosensor based on the Au@Ag@Au NPs and GO dual-amplification strategy was proposed. GO was adsorbed on the surface of an amino-modified gold membrane through electrostatic interactions, and the antibody Ab_1_ against the SARS-CoV-2 N protein was coupled to the surface of the GO-modified sensing chip using EDC to form the Au-MEA-GO/Ab_1_-modified sensing chip. Then, Au@Ag@Au NPs coupled with detection antibody Ab_2_ were used as a signal amplification probe. Then, SARS-CoV-2 N proteins were added to form Au@Ag@Au NPs/Ab_2_/SARS-CoV-2 N protein complexes, which are injected onto the surface of the Au-MEA-GO/Ab_1_-modified sensing chip. Therefore, a sandwich immunoassay based on Au@Ag@Au NPs/Ab_2_/SARS-CoV-2 N protein/Ab_1_ was established for the sensitive detection of SARS-CoV-2 N protein, with a linear detection range of 0.1 ng/mL~1000 ng/mL and LOD of 0.083 ng/mL. The developed method has the merits of good sensitivity, strong anti-interference capability and specificity to provide a reference for the development of multiple highly sensitive detection methods for coronaviruses.

## Figures and Tables

**Table 1:** Comparison of other methods for the detection of SARS-CoV-2.

Analytical Method	Target Analyte	Linear Range	Limit of Detection	Reference
SPR sensor based on Molecularly Imprinted Polymer Nanoparticles amplification strategy	SARS-CoV-2 Virus	0.25~1.75 × 10^6^ particles mL^−1^	3.15 × 10^4^ virus particles	[41]
SPR on the basis of visual color change of the RNA biosensor	SARS-CoV-2 RNA	25 nM~200 nM	0.12 nM	[42]
Photonic crystal fiber (PCF)-based SPR sensor	SARS-CoV-2 Virus	N/A	6.42 × 10^−9^ RIU^2^/nm	[43]
SPR sensor based on large gold nanoparticles	SARS-CoV-2 N protein	N/A	85 fM	[44]
SPR sensor based on Au@Ag@Au NPs and GO double-amplification strategy	SARS-CoV-2 N protein	0.1~1000 ng/mL	0.083 ng/mL	This work

**Table 2:** Detection of SARS-CoV-2 N protein in real samples.

Sample	Added SARS-CoV-2 N protein (ng/mL)	Test (ng/mL)	Recovery (%)	RSD (%)
Artificial saliva	0.2	0.23	116.83	3.88
	100	111.13	111.13	2.66
	400	398.67	99.67	1.50

## Data Availability

Not applicable.

## Supplementary Materials

The following supporting information can be downloaded at: https://www.mdpi.com/article/10.3390/bios13050549/s1, Figure S1: Schematic diagram of SPR sensing device; Table S1: Selection of optimal Ab_2_ concentrations.

## References

[1] Xi H., Jiang H., Juhas M., Zhang Y. (2021). Multiplex Biosensing for Simultaneous Detection of Mutations in SARS-CoV-2. ACS Omega.

[2] Pan P., Shen M., Yu Z., Ge W., Chen K., Tian M., Xiao F., Wang Z., Wang J., Jia Y. (2021). SARS-CoV-2 N protein promotes NLRP3 inflammasome activation to induce hyperinflammation. Nat. Commun..

[3] Anderson G.P., Liu J.L., Esparza T.J., Voelker B.T., Hofmann E.R., Goldman E.R. (2021). Single-Domain Antibodies for the Detection of SARS-CoV-2 Nucleocapsid Protein. Anal. Chem..

[4] Guo A., Pei F., Feng S., Hu W., Zhang P., Xia M., Mu X., Tong Z., Wang F., Liu B. (2023). A photoelectrochemical immunosensor based on magnetic all-solid-state Z-scheme heterojunction for SARS-CoV-2 nucleocapsid protein detection. Sens. Actuators B Chem..

[5] Liu D., Ju C., Han C., Shi R., Chen X., Duan D., Yan J., Yan X. (2020). Nanozyme chemiluminescence paper test for rapid and sensitive detection of SARS-CoV-2 antigen. Biosens. Bioelectron..

[6] Liang C., Liu B., Li J., Lu J., Zhang E., Deng Q., Zhang L., Chen R., Fu Y., Li C. (2021). A nanoenzyme linked immunochromatographic sensor for rapid and quantitative detection of SARS-CoV-2 nucleocapsid protein in human blood. Sens. Actuators B Chem..

[7] Grant B.D., Anderson C.E., Williford J.R., Alonzo L.F., Glukhova V.A., Boyle D.S., Weigl B.H., Nichols K.P. (2020). SARS-CoV-2 Coronavirus Nucleocapsid Antigen-Detecting Half-Strip Lateral Flow Assay Toward the Development of Point of Care Tests Using Commercially Available Reagents. Anal. Chem..

[8] Bialobrzeska W., Ficek M., Dec B., Osella S., Trzaskowski B., Jaramillo-Botero A., Pierpaoli M., Rycewicz M., Dashkevich Y., Łęga T. (2022). Performance of electrochemical immunoassays for clinical diagnostics of SARS-CoV-2 based on selective nucleocapsid N protein detection: Boron-doped diamond, gold and glassy carbon evaluation. Biosens. Bioelectron..

[9] Liang R.P., Yao G.H., Fan L.X., Qiu J.D. (2012). Magnetic Fe_3_O_4_@Au composite-enhanced surface plasmon resonance for ultrasensitive detection of magnetic nanoparticle-enriched alpha-fetoprotein. Anal. Chim. Acta.

[10] Park J.H., Byun J.Y., Mun H., Shim W.B., Shin Y.B., Li T., Kim M.G. (2014). A regeneratable, label-free, localized surface plasmon resonance (LSPR) aptasensor for the detection of ochratoxin A. Biosens. Bioelectron..

[11] Zhang C., Li Z., Jiang S.Z., Li C.H., Xu S.C., Yu J., Li Z., Wang M.H., Liu A.H., Man B.Y. (2017). U-bent fiber optic SPR sensor based on graphene/AgNPs. Sens. Actuators B Chem..

[12] Fathi F., Rashidi M.R., Omidi Y. (2019). Ultra-sensitive detection by metal nanoparticles-mediated enhanced SPR biosensors. Talanta.

[13] Dai Z., Xu X., Wang Y., Li M., Zhou K., Zhang L., Tan Y. (2022). Surface plasmon resonance biosensor with laser heterodyne feedback for highly-sensitive and rapid detection of COVID-19 spike antigen. Biosens. Bioelectron..

[14] Patil P.O., Pandey G.R., Patil A.G., Borse V.B., Deshmukh P.K., Patil D.R., Tade R.S., Nangare S.N., Khan Z.G., Patil A.M. (2019). Graphene-based nanocomposites for sensitivity enhancement of surface plasmon resonance sensor for biological and chemical sensing: A review. Biosens. Bioelectron..

[15] Yi R.M., Zhang Z., Liu C.X., Qi Z.M. (2020). Gold-silver alloy film based surface plasmon resonance sensor for biomarker detection. Mater. Sci. Eng. C Mater. Biol. Appl..

[16] Asadiyan M.H., Parhoodeh S., Nazem S. (2022). Simulation and fabrication of butane gas sensor based on surface plasmon resonance phenomenon. Optik.

[17] Fan L., Du B., Pei F., Hu W., Guo A., Xie Z., Liu B., Tong Z., Mu X., Tan W. (2023). Surface Plasmon Resonance Sensor Based on Core-Shell Fe(3)O(4)@SiO(2)@Au Nanoparticles Amplification Effect for Detection of T-2 Toxin. Sensors.

[18] McCoy G.R., McNamee S., Campbell K., Elliott C.T., Fleming G.T., Raine R. (2014). Monitoring a toxic bloom of Alexandrium minutum using novel microarray and multiplex surface plasmon resonance biosensor technology. Harmful Algae.

[19] Dong T., Han C., Jiang M., Zhang T., Kang Q., Wang P., Zhou F. (2022). A Four-Channel Surface Plasmon Resonance Sensor Functionalized Online for Simultaneous Detections of Anti-SARS-CoV-2 Antibody, Free Viral Particles, and Neutralized Viral Particles. ACS Sens..

[20] Akib T.B.A., Mou S.F., Rahman M.M., Rana M.M., Islam M.R., Mehedi I.M., Mahmud M.A.P., Kouzani A.Z. (2021). Design and Numerical Analysis of a Graphene-Coated SPR Biosensor for Rapid Detection of the Novel Coronavirus. Sensors.

[21] Das C.M., Guo Y., Yang G., Kang L., Xu G., Ho H.P., Yong K.T. (2020). Gold Nanorod Assisted Enhanced Plasmonic Detection Scheme of COVID-19 SARS-CoV-2 Spike Protein. Adv. Theory Simul..

[22] Chen R., Kan L., Duan F., He L., Wang M., Cui J., Zhang Z., Zhang Z. (2021). Surface plasmon resonance aptasensor based on niobium carbide MXene quantum dots for nucleocapsid of SARS-CoV-2 detection. Mikrochim. Acta.

[23] Wu Q., Wu W., Chen F., Ren P. (2022). Highly sensitive and selective surface plasmon resonance biosensor for the detection of SARS-CoV-2 spike S1 protein. Analyst.

[24] Uddin S.M.A., Chowdhury S.S., Kabir E. (2021). Numerical Analysis of a Highly Sensitive Surface Plasmon Resonance Sensor for SARS-CoV-2 Detection. Plasmonics.

[25] Wu Q., Sun Y., Zhang D., Li S., Wang X., Song D. (2016). Magnetic field-assisted SPR biosensor based on carboxyl-functionalized graphene oxide sensing film and Fe_3_O_4_-hollow gold nanohybrids probe. Biosens. Bioelectron..

[26] Chen H., Qi F., Zhou H., Jia S., Gao Y., Koh K., Yin Y. (2015). Fe_3_O_4_@Au nanoparticles as a means of signal enhancement in surface plasmon resonance spectroscopy for thrombin detection. Sens. Actuators B Chem..

[27] Zhang H., Sun Y., Wang J., Zhang J., Zhang H., Zhou H., Song D. (2012). Preparation and application of novel nanocomposites of magnetic-Au nanorod in SPR biosensor. Biosens. Bioelectron..

[28] Grzeskowiak B.F., Tusnio K., Wozniak A., Szalata M., Lipiński D., Jurga S., Słomski R. (2019). Transgenic Plant Detection Using an AuNPs Based SPR Biosensor. Biosensors.

[29] Nain R., Dobhal S., Bidaliya P., Saini G., Pani B., Sirohi S. (2018). Ag decorated silica nanostructures for surface plasmon enhanced photocatalysis. RSC Adv..

[30] Gu Y., Song J., Li M.X., Zhang T., Zhao W., Xu J., Liu M., Chen Y. (2017). Ultrasensitive MicroRNA Assay via Surface Plasmon Resonance Responses of Au@Ag Nanorods Etching. Anal. Chem..

[31] Zhang J., Wu C., Yuan R., Huang J.A., Yang X. (2022). Gap controlled self-assembly Au@Ag@Au NPs for SERS assay of thiram. Food Chem..

[32] Xing L., Xiahou Y., Zhang X., Du W., Zhang P., Xia H. (2022). Large-Area Monolayer Films of Hexagonal Close-Packed Au@Ag Nanoparticles as Substrates for SERS-Based Quantitative Determination. ACS Appl. Mater. Interfaces.

[33] Yao L.H., Zhang J.P., Dai H.W., Wang M.S., Zhang L.M., Wang X., Han J.B. (2018). Plasmon-enhanced versatile optical nonlinearities in a Au-Ag-Au multi-segmental hybrid structure. Nanoscale.

[34] Ye X., Shi H., He X., Wang K., Li D., Qiu P. (2014). Gold nanorod-seeded synthesis of Au@Ag/Au nanospheres with broad and intense near-infrared absorption for photothermal cancer therapy. J. Mater. Chem. B.

[35] Wei W., Nong J., Mei Y., Zhong C., Lan G., Hu W. (2018). Single-layer graphene-coated gold chip for enhanced SPR imaging immunoassay. Sens. Actuators B Chem..

[36] Chiu N.F., Fan S.Y., Yang C.D., Huang T.Y. (2017). Carboxyl-functionalized graphene oxide composites as SPR biosensors with enhanced sensitivity for immunoaffinity detection. Biosens. Bioelectron..

[37] Wang Q., Wang B.-T. (2018). Surface plasmon resonance biosensor based on graphene oxide/silver coated polymer cladding silica fiber. Sens. Actuators B Chem..

[38] Wei D., Zhang X., Chen B., Zeng K. (2020). Using bimetallic Au@Pt nanozymes as a visual tag and as an enzyme mimic in enhanced sensitive lateral-flow immunoassays: Application for the detection of streptomycin. Anal. Chim. Acta.

[39] Li J.F., Tian X.D., Li S.B., Anema J.R., Yang Z.L., Ding Y., Wu Y.F., Zeng Y.M., Chen Q.Z., Ren B. (2013). Surface analysis using shell-isolated nanoparticle-enhanced Raman spectroscopy. Nat. Protoc..

[40] Fales A.M., Yuan H., Vo-Dinh T. (2014). Development of Hybrid Silver-Coated Gold Nanostars for Nonaggregated Surface-Enhanced Raman Scattering. J. Phys. Chem. C Nanomater. Interfaces.

[41] Bajaj A., Trimpert J., Abdulhalim I., Altintas Z. (2022). Synthesis of Molecularly Imprinted Polymer Nanoparticles for SARS-CoV-2 Virus Detection Using Surface Plasmon Resonance. Chemosensors.

[42] Jamaluddin N.D., Ibrahim N., Yusof N.Y.M., Goh C.T., Tan L.L. (2023). Optical reflectometric measurement of SARS-CoV-2 (COVID-19) RNA based on cationic cysteamine-capped gold nanoparticles. Opt. Laser Technol..

[43] Islam A., Haider F., Ahmmed Aoni R., Ahmed R. (2022). Plasmonic photonic biosensor: In situ detection and quantification of SARS-CoV-2 particles. Opt. Express.

[44] Yano T.A., Kajisa T., Ono M., Miyasaka Y., Hasegawa Y., Saito A., Otsuka K., Sakane A., Sasaki T., Yasutomo K. (2022). Ultrasensitive detection of SARS-CoV-2 nucleocapsid protein using large gold nanoparticle-enhanced surface plasmon resonance. Sci. Rep..

