# Supplementary material for: A Novel SPR Immunosensor Based on Dual Signal Amplification Strategy for Detection of SARS-CoV-2 Nucleocapsid Protein
Source: Biosensors (Basel). 2023 May 15;13(5):549. doi: 10.3390/bios13050549 (PMC10216089; doi:10.3390/bios13050549)
Supplement: Supplementary file 1 [file biosensors-13-00549-s001.zip › biosensors-2371922-supplementary.pdf]

Supplementary materials

A Novel SPR Immunosensor Based on Dual Signal Amplification Strategy for Detection of SARS-CoV-2 Nucleocapsid Protein

Lirui Fan [1,2], Bin Du [1], Fubin Pei [1], Wei Hu [1], Shasha Feng [1], Bing Liu [1], Zhaoyang Tong [1], Wenyuan Tan [2,*] and Xihui Mu [1,*]

[1] State Key Laboratory of NBC Protection for Civilian, Beijing 102205, China;
[2] School of Chemical Engineering, Sichuan University of Science and Engineering, Zigong 643000, China
* Correspondence: twyhyx@126.com (W.T.); mxh0511@sohu.com (X.M.)

2.1. Reagents and Instruments

N protein of influenza A (Flu A), hemagglutinin of influenza B (Flu B), respiratory syncytial virus attachment protein G (RSV), N protein of middle east respiratory syndrome coronavirus (MERS), monoclonal antibody (Ab2, Ab1) against SARS-CoV-2 N protein and SARS-CoV-2 N protein were purchased from Shandong Landu Biotechnology Co., Ltd. Diiodomethane (n=1.74) and monolayer graphene oxide were purchased from Beijing Bailingwei Technology Co., Ltd. β-mercaptoethylamine (MEA) and ethanolamine hydrochloride (EA) were purchased from Shanghai Aladdin Biochemical Technology Co., Ltd. N-hydroxysuccinimide (NHS), 1-ethyl-3-(3- dimethylaminoropyl) carbo-diimide hydrochloride (EDC) and bovine serum albumin (BSA) were purchased from Sigma Company (China). PBS buffer solution, PBST buffer solution (0.1% Tween 20%) and ultrapure water are all made by our laboratory.

The zeta potential data of Au NPs, Au@Ag NPs and Au@Ag@Au NPs were accessed using a Zetasizer Nano-ZS nano analyzer (Malvern, UK). Determination of the UV-Vis absorption spectra was performed using an Ultramicro Nucleic Acid Protein Quantitative Instrument (Thermo Fisher, USA). The AFM images were obtained by an automatic probe scanning microscope (NT-MDT, Russia).

2.2. Construction of the SPR sensor

The SPR sensor used in this work was constructed by panelists. The SPR sensor is constructed using the Kretschmann structure as the SPR sensing model, and the surface plasmon excitation effect was excited by attenuated total reflection (ATR). Additionally, the SPR sensor obtains continuous wavelengths from a tungsten halogen lamp (LS-1, Ocean Optics, USA), which are passed through a quartz fiber, collimator and linear polarizer to form *p* polarized parallel light. Then, the p-polarized parallel light is incident on the prism at a fixed angle of incidence (θ = 13°). The reflectivity curve of the wavelength versus reflected light is detected at the interface of the prism-coupled metal film. The wavelength corresponding to the lowest reflectivity of the reflected light is obtained as the resonance wavelength (λ). Finally, the reflected beam is transmitted to

a spectrometer (USB 2000+, Ocean Optics, USA) through a collimator and quartz fiber, and the spectral data are recorded. In particular, the gold chip was placed on an optical trigon (45°/45°/90°, n=1.799 at 632.8 nm, Scott N-SF6) and fixed on the SPR prism using a sample chamber. Diiodomethane (n=1.74) was added dropwise to enhance the coupling between the prism and the sensing chip. Finally, the sample chamber, the gold chip and the prism were fixed on the SPR prism to form the sandwich structure shown in Figure S1, forming the SPR sensing detection platform.

The device obtains information on the binding between the receptor molecule and the ligand molecule by measuring the amount of change in the position of the resonance peak

in the SPR spectrum. When SPR resonance is generated, the position of the resonance peak is related to the refractive index of the metal layer surface medium, which varies with the mass of the attached biomolecule. In the SPR sensing assay, the ligand is coupled to the surface of the gold chip to form a functionalized sensing gold chip, and the sample containing the analyte is injected onto the surface of the functionalized chip, where the binding of the ligand to the analyte causes a change in the refractive index of the surface of the sensing chip and a consequent change in the resonance peak.

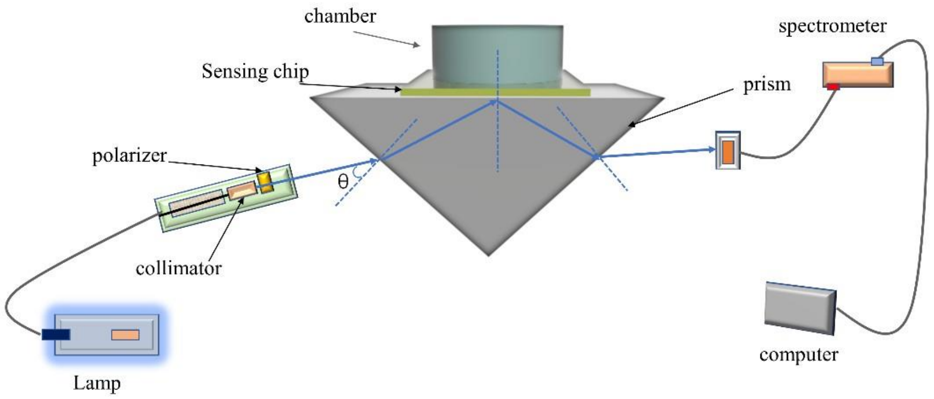

Figure S1. Schematic diagram of SPR sensing device.

Table S1. Selection of optimal Ab2 concentrations.

Concentration of Ab2 (ng/mL)	Ultraviolet Absorption of Supernatant (280 nm)
50	0
100	0.005
200	0.008
300	0.056
400	0.140
